# Geological support for the Umbrella Effect as a link between geomagnetic field and climate

**DOI:** 10.1038/srep40682

**Published:** 2017-01-16

**Authors:** Ikuko Kitaba, Masayuki Hyodo, Takeshi Nakagawa, Shigehiro Katoh, David L. Dettman, Hiroshi Sato

**Affiliations:** 1 Research Centre for Palaeoclimatology, Ritsumeikan University, Kusatsu 525-8577, Japan; 2 Research Center for Inland Seas, Kobe University, Kobe 657-8501, Japan; 3 Museum of Nature and Human Activities, Hyogo, Sanda 669-1546, Japan; 4 Department of Geosciences, University of Arizona, Tucson, AZ 85721, USA; 5 Institute of Natural and Environmental Sciences, University of Hyogo, Sanda 669-1546, Japan

## Abstract

The weakening of the geomagnetic field causes an increase in galactic cosmic ray (GCR) flux. Some researchers argue that enhanced GCR flux might lead to a climatic cooling by increasing low cloud formation, which enhances albedo (umbrella effect). Recent studies have reported geological evidence for a link between weakened geomagnetic field and climatic cooling. However, more work is needed on the mechanism of this link, including whether the umbrella effect is playing a central role. In this research, we present new geological evidence that GCR flux change had a greater impact on continental climate than on oceanic climate. According to pollen data from Osaka Bay, Japan, the decrease in temperature of the Siberian air mass was greater than that of the Pacific air mass during geomagnetic reversals in marine isotope stages (MIS) 19 and 31. Consequently, the summer land-ocean temperature gradient was smaller, and the summer monsoon was weaker. Greater terrestrial cooling indicates that a reduction of insolation is playing a key role in the link between the weakening of the geomagnetic field and climatic cooling. The most likely candidate for the mechanism seems to be the increased albedo of the umbrella effect.

Debate continues over whether changes in the galactic cosmic ray (GCR) flux may induce climate change. Weakening of the geomagnetic field and its shielding properties leads to an increase in GCR flux, and this increased GCR may induce more active low cloud formation (Svensmark effect)^1^, which in turn increases albedo and hence reduces temperature^2^. Two possible mechanisms have been proposed for how GCR affects clouds^3^: one theory argues that the highly-charged GCR ionises a number of atmospheric molecules, leading to combination and nucleation of aerosol particles which grow into cloud condensation nuclei (CCN)^4^,^5^,^6^,^7^,^8^; the other proposes that GCR affects clouds through the modulation of the global electric circuit^9^.

Interest is strong in proving or disproving the Svensmark effect as an important factor affecting climate because, if the hypothesis is correct, it would comprise an important mechanism in which solar variation may control climate through the modulation of GCR flux^10^.

Significant GCR change, however, occurs rarely and on geological time scales and is not suitable for direct observation. Therefore, the GCR linkage to climate change is accordingly difficult to assess within a manageable time frame. Numerous experiments and modelling studies have examined the production and growth of cloud condensation nuclei by the atmospheric ionisation generated by GCR^4^,^5^,^6^,^7^,^8^. These studies, however, have been inconclusive because the physical scale and complexity of this process is too large to be reproduced experimentally or in model simulation.

Geomagnetic polarity reversals, which occur infrequently on a geological time scale (a few times in 10^6^ years), provide a solution to this problem. During geomagnetic polarity reversals, the geomagnetic field intensity drops to 10–20% of the present value, which increases the GCR by 90–70%^11^. Such conditions persist over thousands of years^12^,^13^, permitting geologists to assess how the climate responded to the GCR change over that period. Recent palaeoclimatic research has revealed that geomagnetic polarity reversals coincided with climatic cooling. Two anomalous cooling events are observed during the Matuyama‒Brunhes and Lower Jaramillo geomagnetic reversals, which occurred ca. 780 ka and 1,070 ka, respectively, in palaeoclimatic records from Osaka Bay, southwest Japan^14^,^15^. These cooling events cannot be explained by conventional Milankovitch theory and seem to have occurred across a widespread area in low- and mid-latitudinal regions^14^,^16^, such as southeastern Siberia^17^, Italy^18^,^19^,^20^ and Israel^21^.

Despite all these efforts, however, all that has been observed so far is ‘synchronicity’ between geomagnetic and temperature changes, leaving more detailed linking mechanisms such as the GCR-induced cloud formation and the subsequent reduction of insolation (umbrella effect) still hypothetical. In this study, we present geological data which supports the umbrella effect as the most likely cause of the geomagnetic field-climate link. More specifically, we quantified the magnitude of cooling in both continental and oceanic climates. Because land has a smaller heat capacity than the oceans, continental climates are more sensitive to insolation forcing. Using pollen analysis of a sediment core from Japan, we demonstrate differential cooling in seasonal temperatures, as well as weakening of the East Asian summer monsoon. These observations in combination support the idea that the differences in response to a reduction of insolation caused by cloud formation was a central factor in the mechanism that connects GCR and climate changes.

## Geographical setting

**Figure 1:**
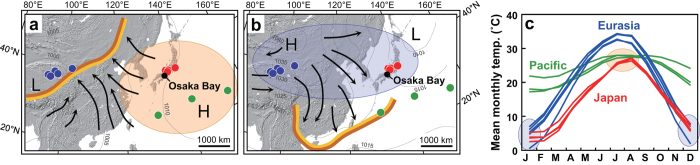
Location map of Osaka Bay and atmospheric circulation system in the East Asian monsoon region. (**a,b**) Atmospheric pressure patterns in summer and winter, and the changes in wind direction. Thick yellow lines show the monsoon front. (**c**) Observed mean temperature of each month for the Eurasian interior (blue), Pacific islands (green) and Japan (red) (modified after ref. 22).

Japan is ideally located for comparison of terrestrial and oceanic temperature change in this context, using quantitative and comparable proxies. In summer, the Japanese climate is controlled by the Pacific air mass (Fig. 1a), whereas it is controlled by the Siberian air mass in winter (Fig. 1b). Due to this geographical setting, Japanese summer and winter temperatures can be used as good indicators of the temperatures of the Pacific and Siberian air masses^22^ (Fig. 1c). In addition, because of the different heat capacities of the land and the ocean, the summer temperature of the Siberian air mass is significantly higher than that of the Pacific air mass (Fig. 1c). This thermal gradient drives the East Asian summer monsoon, in which the dominant wind direction is from the Pacific Ocean to the Eurasian interior (Fig. 1a).

The sediments of the Osaka Basin consist of alternating marine and fluvio-lacustrine sediments that correspond to glacial-interglacial cycles, and hold records of detailed palaeoenvironmental changes since the Late Pliocene^23^. The first seawater intrusion into Osaka Bay is dated at 1.25 Ma, which is in marine oxygen isotope stage (MIS) 37^24^. Since then, Osaka Bay was the locus of marine depositional environments during interglacial periods^23^. During glacial periods it was filled with freshwater from inflowing rivers when the sea-level dropped below the sill between Osaka Bay and the Pacific Ocean^25^. The age-depth model of the sediment core was primarily established by correlating homogeneous marine clay layers to interglacial periods. The chronology was further refined using the magneto- and tephro-stratigraphy of the core^23^,^24^,^26^ (Methods).

**Figure 2:**
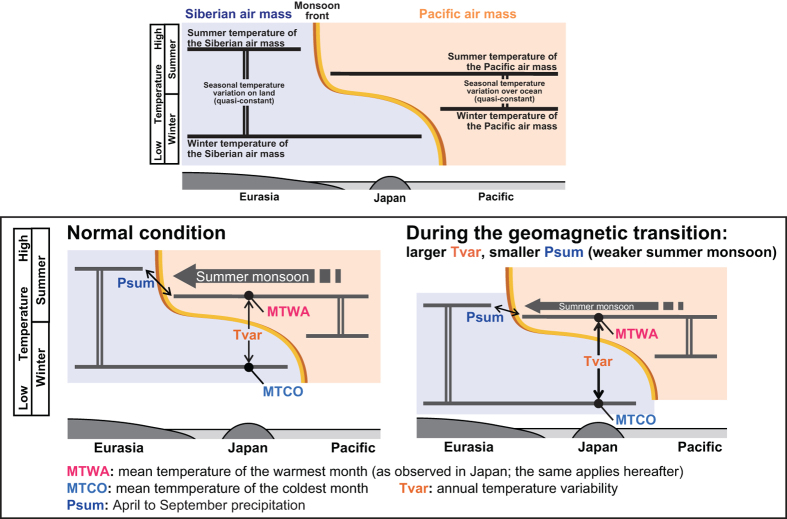
Schematic model of the East Asian monsoon climate system and reconstructed climatic parameters in Japan. Left: Normal condition^22^. MTWA and MTCO are the summer and winter temperatures in Japan, which represent temperatures of the Pacific and Siberian air masses, respectively. Psum, total Japanese summer precipitation, is proportional to the land-ocean temperature gradient in summer. Right: During the geomagnetic transition, both terrestrial and oceanic air temperatures drop, but the cooling effect was larger over land due to its smaller heat capacity. Hence, the land-ocean temperature gradient in summer decreases, weakening the summer monsoon, which then is reflected in decreased summer precipitation in Japan.

Palaeomagnetic and palynological data in centennial- to millennial-resolution are available for MISs 19 and 31^13^,^14^ from the “1,700-m” sediment core from Osaka Bay, Japan (Methods). Those interglacial periods were targeted because they contain palaeomagnetic reversals. Also available for comparison are pollen records from MIS 21 and 25 which are interglacials without geomagnetic polarity reversal^14^,^27^. All pollen data were converted into quantitative climate indices using the modern analogue technique which has been well established in the region^22^,^28^,^29^ (Methods). The reconstructed indices are summer temperature (MTWA; defined as the mean temperature of the warmest month), winter temperature (MTCO; defined as the mean temperature of the coldest month), and summer precipitation (Psum; defined as the cumulative precipitation from April to September). Previous studies have demonstrated that these indices (MTWA, MTCO and Psum) in Japan can be used as proxies of the temperature of the Pacific air mass, temperature of the Siberian air mass, and the East Asian summer monsoon intensity, respectively^22^,^29^ (Fig. 2, left panel).

From this schematic picture of the Asian monsoon system one would expect an anti-correlation between summer monsoon strength and annual temperature variability in Japan. This anti-correlation is indeed observed in the palaeoclimate records from Lake Biwa for the last 450 kyrs—throughout the entirety of the available record^22^. Also predicted from the same schematic model is the anti-correlation between summer and winter monsoon intensities. This has not been demonstrated in the records from Lake Biwa^22^ or Osaka Bay as they are both located on the Pacific side of the archipelago (i.e. on the southern side of main watershed) and the moist wind from the Sea of Japan cannot reach those regions. However, the summer monsoon intensity from Hulu and Dongge caves in China^30^,^31^ and the winter monsoon (driven by the land-ocean temperature gradient in winter) intensity from Lake Huguang Maar, southeast China also show a clear inverse correlation over the period of 9–15 cal. kyr BP^32^. Short-term annual/seasonal climate is, of course, subject to a number of local and temporary factors. However, the evidence cited above supports the use of long term averages of seasonal climate parameters (millennial to orbital scales) for the island of Japan as a tool to reconstruct Pacific and Eurasian climates separately.

## Results and Discussion

**Figure 3:**
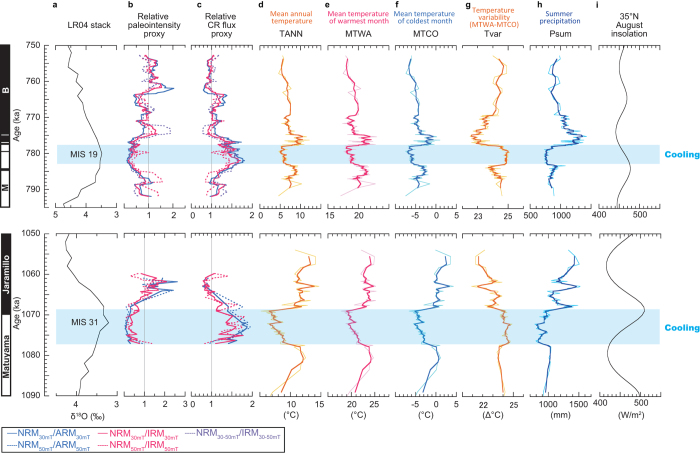
Geomagnetic field variation and climate change during MISs 19 and 31. In the magneto-stratigraphy, black/white denotes normal/reverse polarities. (**a**) Marine oxygen isotope stack LR04^34^. (**b**) Relative magnetic palaeointensity. The present value is set to 1. Palaeomagnetic data for Matuyama-Brunhes and Lower Jaramillo are from refs 13 and 14. Upper and lower sections of the MIS 31 were not suitable for palaeomagnetic measurements because of coarser sediments^14^. (**c**) Relative cosmogenic radionuclide production rate^11^,^14^ calculated from geomagnetic field intensity data (**b**). (**d–h**) Reconstructed climatic parameters shown in Fig. 2. (**i**) August insolation at 35°N^46^.

The reconstructed MTWA and MTCO both show that the anomalous cooling events coincide in time with the low magnetic field (i.e. high GCR flux) intervals of the geomagnetic polarity reversals (Fig. 3a–f). Both cooling events occurred when the thermal maximum would be expected if the system simply follows Milankovitch orbital forcing. The cooling events lasted ~5,000 yrs (783–778 ka) in MIS 19 and ~8,000 yrs (1,077–1,069 ka) in MIS 31. If we look at the amplitude of the change, the total decrease was different between MTWA and MTCO. In MIS 19, MTWA decreased by ca. 2 °C whereas MTCO decreased by ca. 3 °C: the temperature decrease in winter was larger than summer. Also in MIS 31, the MTWA and MTCO went down by ca. 2–4 °C and ca. 3–5 °C, respectively. In other words, annual temperature variability (Tvar: defined as MTWA minus MTCO) became larger (Fig. 3g). In both periods, the Psum decreased by ca. 100–200 mm (Fig. 3h).

**Figure 4:**
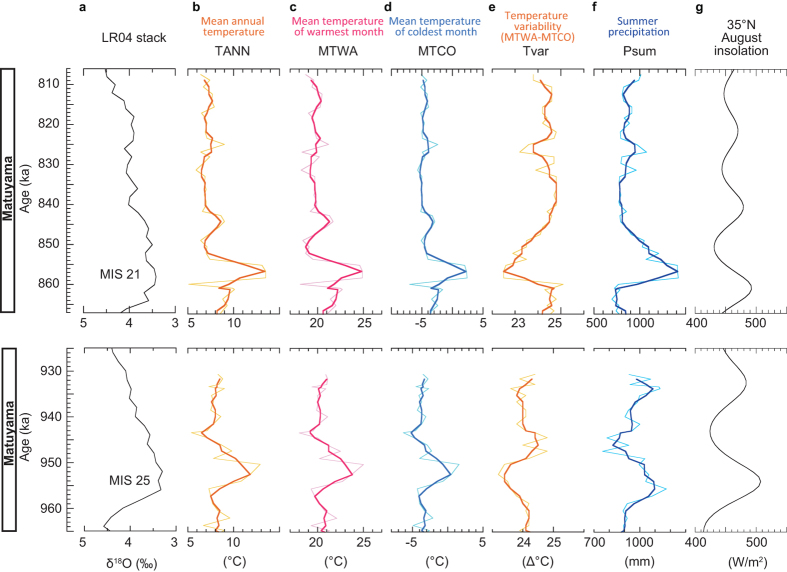
Climate change during MISs 21 and 25. (**a**) Marine oxygen isotope stack LR04^34^. (**b**–**f**) Reconstructed climatic parameters shown in Fig. 2. (**g**) August insolation at 35°N^46^.

In contrast, the temperature and precipitation in MIS 21 and 25 vary following boreal summer insolation as predicted by Milankovitch orbital forcing (Fig. 4). Mean annual temperature (TANN), MTWA and MTCO in MIS 21 has multiple peaks that correspond to summer insolation maxima. Those peaks also correspond very well to the precessional oscillations in the low latitude marine δ^18^O stack^33^. The corresponding peaks are not strongly pronounced in the LR04 stack^34^, which is most likely an artifact of stacking a large number of curves including some low-resolution records. In MIS 25, TANN, MTWA and MTCO follow the LR04 stack^34^ (the low latitude δ^18^O stack^33^ is not available at this age) within the correlation error (Method). During the sea-level highstands of MIS 21 and 25, Tvar decreased by ca. 2 and 1 °C and Psum increased by ca. 600–700 mm and 200 mm, respectively. These observations about interglacials without magnetic reversals uniformly support the widely accepted theory that insolation regulates temperature and monsoon intensity^22^,^30^,^32^,^35^,^36^,^37^,^38^. During geomagnetic reversals (MISs 19 and 31), however, the response is very different and another forcing factor must exist.

Although the differences in the reconstructed cooling between seasons are relatively small, we are confident that they are nonetheless real, as the pattern is similar in both cooling episodes MIS 19 and MIS 31 (Fig. 3d–g), and both are accompanied by a reduction of Psum, which indicates a weakening of the summer monsoon (Fig. 3h). The summer monsoon is primarily driven by the land-ocean temperature gradient (Fig. 2, left panel). If the amplitude of cooling were identical for land and ocean, then cooling would not induce a change in the gradient (or in monsoon intensity and precipitation). The gradient changes when the cooling of one air mass is greater than the other (Fig. 2, right panel). The observed decrease of summer precipitation, therefore, provides objective support for a stronger cooling of the continental air mass (i.e. a larger temperature drop in Japanese winter than in summer) during the period of the geomagnetic polarity reversal. Unlike many other methods, climate indices reconstructed by the modern analogue technique are essentially independent from each other, which means that the coupling of the temperature and precipitation change is not an artifact of the reconstruction method. In Lake Biwa, which is very near Osaka Bay (our core site), temperature and precipitation inferred from pollen data using the same method as this study are indeed tracking different orbital rhythms (eccentricity and precession, respectively) for the last 450 kyrs^22^, proving that the thermal and hydrological parameters are independently reconstructed.

The bias of cooling toward land mass over ocean, supported by evidence of the weakening of the summer monsoon, requires a mechanism which has greater effect on land than ocean, and the most likely cause is the different heat capacities of land masses and the ocean regions which generates differential responses to the umbrella effect. If a sequence of climate change during the geomagnetic reversals is attributed to the GCR-cloud effect^1^, the albedo would become larger in these periods. The resulting reduced insolation leads to greater cooling of objects with smaller heat capacity, which would be terrestrial regions in the case of the land-ocean temperature balance. A supporting example of increased albedo is the Toba super eruption where the cooling of land masses was significantly greater than that of oceans^39^. Because smaller heat capacity is a simple physical property of land that is not specific to any region, the observed cooling may have occurred in a much wider geographical area.

Another implication of our findings is that there is an additional mechanism affecting variation in monsoon strength. It has long been understood that orbital forcing is the key to understand monsoon rhythms^22^,^30^,^35^,^36^,^37^,^38^. Change in GCR flux may also affect monsoon variation on a yet different timescale. Because Osaka Bay does not yield continuous fine-grained sediments from glacial periods, we can only examine the coupling of the geomagnetic field intensity and monsoon climate during interglacial periods. In the δ^18^O records of the Chinese speleothems that have been widely accepted as a high-resolution template of Asian summer monsoon intensity changes^30^,^35^,^38^, there does not seem to be any visible weakening of monsoon signals during magnetic excursions. This could be because the effects of excursions were more short-lived and weaker when compared with magnetic reversals. One exception to this rule could be the Laschamp excursion during which the magnitude of the weakening of the magnetic field was comparable to that of the reversals^40^,^41^. Unfortunately, however, the climate recorded in glacial interval Chinese speleothems was already dry and cold with enhanced millennial scale oscillations, which combine to make detection of additional temperature cooling and monsoon weakening very difficult. Whether the Svensmark effect drives monsoon climate persistently through glacial-interglacial cycles or it is restricted to the interglacial periods remains unknown.

## Methods

### Pollen analysis and pollen-based climate reconstruction.

Samples were taken from the “1,700-m core” which was taken from northeastern Osaka Bay in 1996 by the wire-line method^42^ with a recovery rate of about 96%^24^. The 1,700-m core penetrated the granitic basement rock at 1,545.5-m depth. It recovered a long series of marine fine clay to silty clay beds and fluvio-lacustrine silts, sands and gravels, representing glacial – interglacial cycles^24^,^26^. The age-depth model of this core was primarily established by correlating the homogeneous marine clay units to the interglacial periods, and then further refined by the magneto- and tephro-stratigraphy^23^,^24^,^26^.

A total of 271 samples were taken in and around the marine clay layers corresponding to MISs 19, 21, 25 and 31. Samples were spaced by ca. 200 yrs around the geomagnetic transition and by ca. 1,600 yrs for other periods. Each sample was 2-cm thick, representing ca. 32 yrs. Pollen fossils were extracted by the standard chemical–physical procedures^27^: 10% KOH, rinsing with water, heavy liquid separation with ZnCl_2_ (s.g. 2.0), 25% HF and acetolysis method. At least 300 (407 in average) arboreal pollen grains were counted under the optical microscope with the magnification of 400x. The complete dataset is available in ref. 14.

The quantitative climate reconstruction was performed by the modern analogue technique^28^. Thirty two major pollen taxa^43^ were used for the reconstruction. Polygon 2.4.3 software (http://polsystems.rits-palaeo.com/) and associated 421 modern pollen and 147 climate datasets were used for the climate reconstruction. Eight modern pollen spectra, which have the smallest chord distance in the 32-dimention Euclidian space were selected as the best modern analogues for each fossil pollen spectrum. The climatic indices were estimated by averaging eight best modern analogues using the chord distance as the weighting factor^28^. The accuracy of TANN, MTWA, MTCO and Psum reconstructions was consistently high with r value of 0.851, 0.832, 0.854 and 0.819, respectively.

### Age-depth model

Details of the age-depth model are presented in ref. 14. Since oxygen isotope data cannot be obtained from the 1,700-m core due to the absence of foraminifera fossils^44^, diatom data from the core^13^,^14^,^27^ were used to reconstruct sea-level variations and the detected sea-level highstands were calibrated to the minima of the LR04 marine oxygen isotope (δ^18^O) stack^34^. The estimated error in relative age (correlation error between Osaka Bay and the LR04) is 2.3 ka for MIS 19, 3.6 ka for MIS 21, 6.4 ka for MIS 25 and 2.2 ka for MIS 31. The correlation uncertainty mainly comprises isotope stack’s^34^ relatively low data interval (2 kyrs) as well as its multi-modal nature during interglacials.

### Estimation of relative palaeogeomagnetic intensity and GCR flux proxies

Palaeomagnetic data from the 1,700-m core is also available for MISs 19 and 31 with temporal resolutions of ca. 250 and 350 yrs, respectively^13^,^14^. The half-lock in depth for magnetization is estimated to be less than 10 cm for marine clay in Osaka Bay^45^. Such a small half-lock in depth enables us to assess the relationship between climatic and palaeomagnetic events from the same core. Relative palaeointensity was calculated using the same method used in ref. 13. For the Matuyama‒Brunhes geomagnetic reversal, four proxies are estimated^13^: natural remanent magnetization (NRM) intensity values demagnetized at 30 mT were normalized by anhysteretic remanent magnetization (ARM) demagnetized at 30 mT (NRM_30mT_/ARM_30mT_); NRM intensity values demagnetized at 30 and 50 mT were normalized by isothermal remanent magnetization (IRM) intensity values demagnetized at 30 mT and 50 mT, respectively (NRM_30mT_/IRM_30mT_ and NRM_50mT_/IRM_50mT_); the difference between NRM intensity values demagnetized at 30 and 50 mT divided by the difference between IRM intensity values demagnetized at 30 and 50 mT (NRM_30–50mT_/IRM_30–50mT_). For the Lower Jaramillo reversal, NRM intensity values demagnetized at 50 mT were normalized by ARM demagnetized at 50 mT (NRM_50mT_/ARM_50mT_) were used instead of NRM_30–50mT_/IRM_30–50mT_; the other three proxies are the same^14^.

Relative GCR flux modulated by the geomagnetic field is calculated as the global average production rate of ^10^Be where the long-term average solar modulation parameter ϕ = 550 MeV^11^. The error is estimated to be 7%^11^.

## Additional Information

**How to cite this article**: Kitaba, I. *et al*. Geological support for the Umbrella Effect as a link between geomagnetic field and climate. *Sci. Rep.* **7**, 40682; doi: 10.1038/srep40682 (2017).

**Publisher's note:** Springer Nature remains neutral with regard to jurisdictional claims in published maps and institutional affiliations.
